# Editorial: Open science in consciousness research

**DOI:** 10.1093/nc/niz018

**Published:** 2019-12-13

**Authors:** Anil K Seth, Jakob Hohwy

**Affiliations:** 1 Sackler Centre for Consciousness Science, School of Engineering and Informatics, University of Sussex, Brighton, BN1 9QJ, UK and Canadian Institute for Advanced Research, Azrieli Programme on Brain, Mind, and Consciousness; 2 Department of Philosophy, Monash University, Cognition & Philosophy Lab 20 Chancellor's Walk, RmE672, Melbourne, VIC, Australia

All of us involved in the mind and brain sciences, in whatever capacity, are increasingly aware of the importance of ensuring the credibility of our research. This credibility—which for experimental work turns on its reliability—is particularly salient for consciousness research, given the at-times precarious perception of consciousness science within the wider landscapes of psychology and neuroscience (Michel *et al.* 2019). We are therefore very pleased to introduce a number of ‘open science’ initiatives that have recently been implemented in *Neuroscience of Consciousness*.

One key objective of open science is to actively resist the tendency to search for, and to preferentially publish, eye-catching findings that fit with compelling narratives. This means supporting studies that have been designed to report unbiased results that are reproducible and replicable, whether or not they are narratively convenient.

The gold standard approach here is the Registered Report (RR) format. Intuitively, the idea is to allow researchers to retain complete control over their hypotheses, methods and interpretations, but not over the results (Hardwicke and Ioannidis 2018; Chambers 2019). RRs divide the peer review process into two stages. In the first, reviewers evaluate the rationale, hypotheses, methods and proposed analyses for a study. These are described in a ‘stage 1’ manuscript. This stage takes place before any data collection, other than pilot data collected purely to inform study design. Submissions can be rejected, sent for revision, or accepted at this stage. Importantly, stage 1 peer review offers opportunities to enhance study design—including correction of potentially fatal confounds and flaws—while there is still time. This benefits the researchers and is rewarding for the reviewers.

Once a stage 1 submission is accepted, it is preregistered in a time-stamped public repository such as the Open Science Framework (https://osf.io/), with or without a temporary embargo. Following this ‘in principle acceptance’, the researchers carry out their planned data collection and perform their preregistered analyses, as well as any additional, clearly demarcated, exploratory analyses. They then submit a stage 2 manuscript incorporating these results and a discussion. The introduction and methods remain unchanged from the stage 1 in-principle-accepted manuscript.

This stage 2 submission is sent back to the original reviewers—who cannot at this point raise *post* *hoc* objections to the study rationale or design. Critically, the journal is committed to publish this stage 2 manuscript no matter how the results turn out, so long as the researchers followed the procedures that they described in their stage 1 submission, and have written up their results and discussion sections satisfactorily, as judged by the reviewers. The wow-factor of the results is simply not relevant to the acceptance decision.

In this way, publication of a RR does not depend on the appeal of a *post hoc* story based on eye-catching findings, but instead on the significance of the research question and the adequacy of the experimental design to address this question. As mentioned, exploratory analyses are not excluded by the RR format. In fact, they are encouraged—so long as they are clearly marked as exploratory and reported separately from the primary preregistered analyses.

*Neuroscience of Consciousness* has been accepting RR submissions since June 2019, and we are delighted that Prof. Zoltán Dienes (University of Sussex, UK) has joined our editorial team specifically to handle these manuscripts. We strongly encourage consciousness researchers to take advantage of this innovative format. The journal webpages contain further information about the format, including detailed instructions on how to prepare RR submissions (https://academic.oup.com/nc/pages/General_Instructions).

Despite the many benefits offered by RRs, both to individual researchers and to the wider community, we recognize that it is not always possible to follow this process to its fullest extent. This may be the case when data collection cannot be timed to the researcher’s convenience; where the research is purely exploratory, or focuses on methodological innovations, or is otherwise constrained in ways that preclude the RR procedure. Some kinds of research—such as computational modelling—fit uneasily in present RR formats.

Fortunately, RRs are not the only tool by which open science can be facilitated. Experimental designs and proposed analyses can still be—and wherever possible should be—preregistered prior to data collection (Nosek *et al.*, 2018). This means that the critical distinction between (preregistered) confirmatory and (non-preregistered) exploratory analyses can still be maintained. As with RRs, this separation mitigates against the selective reporting or ‘cherry picking’ of results, as well as against p-hacking (conducting repeated analyses until a desired outcome is obtained, without sufficient statistical correction) and HARK-ing (hypothesizing-after-the-results-are-known)—all of which constitute seductive traps for non-preregistered research. Several options for preregistration are available. One flexible format is provided by www.aspredicted.org—with the Open Science Framework again providing a suitable repository.

Other open science initiatives focus on transparency. Experimental data can be shared, along with code implementing the methods and statistical analyses—allowing other researchers to regenerate the results. Again, repositories such as the Open Science Framework are ideal for this purpose. Sometimes, of course, data sharing might not be possible—for example if such sharing might breach patient confidentiality in clinical studies.

To recognize the uptake of these important innovations, *Neuroscience of Consciousness* now awards Open Science Badges to qualifying papers. These Badges have been developed by the Center for Open Science (https://cos.io), and there is some evidence that they successfully incentivize open science compliance (Kidwell *et al.* 2016). Each awarded badge features in the published article, enhancing its impact. Three Badges are available for *Neuroscience of Consciousness*: Open Materials, Open Data and Preregistration. A manuscript can be awarded one, two, or all three, if it qualifies. Authors need only submit a brief Open Science Badge application form along with their manuscript, in order to be considered.

These open science initiatives are not limited to interactions among researchers, reviewers and journals. The ecology of scientific research, as a whole, needs to co-evolve. Further back in the timeline, funding decisions should increasingly be informed by the principles of open science so that grants are awarded on the basis of quality of proposed research rather than on narrative appeal. Academic societies, such as our partner society the Association for the Scientific Study of Consciousness (ASSC, www.theassc.org), can play an increasingly important role by supporting and giving prominence to open science initiatives. Appointments and promotions up and down academic hierarchies should place more value on robust research, prioritizing quality over quantity and over sometimes diaphanous notions of ‘impact’. Open science is often ‘slow science’—and this slowness and thoroughness should be recognized and valued (Frith 2019). Finally, media coverage must resist the temptation to overplay underpowered research even when—*especially* when—it tells a nice story.

There is, of course, a place for stories too. In the end, the project of science is to develop a compelling story about how nature works. Open science is not about overturning this ideal. Far from it. Open science is about refining what such stories consist in and how they come to be written. A good scientific story is an empirically robust story.

These are complex and systemic challenges. Their solution will depend on many mechanisms operating at many levels, each serving to better align incentives with desired outcomes. We hope that the open science initiatives now implemented at *Neuroscience of Consciousness* will contribute to this evolution. 
